# Endothelial *GNAQ* p.R183Q mutation confers hemoporfin-mediated photodynamic therapy resistance and drives pathological angiogenesis via the angiopoietin-2/TIE2/PI3K/AKT pathway

**DOI:** 10.3389/fcell.2025.1622961

**Published:** 2025-08-18

**Authors:** Lu Liu, Linmei Wang, Jiaxin Luo, Jiayun Yu, Liang Xie, Yang Liu, Hong Xu, Fan Hu, Hanmin Liu

**Affiliations:** ^1^ Department of Pediatric Pulmonology and Immunology, West China Second University Hospital, Sichuan University, Chengdu, China; ^2^ Key Laboratory of Birth Defect and Related Diseases of Women and Children (Sichuan University), Ministry of Education, Chengdu, China; ^3^ NHC Key Laboratory of Chronobiology (Sichuan University), Chengdu, China; ^4^ The Joint Laboratory for Lung Development and Related Diseases of West China Second University Hospital,Sichuan University and School of Life Sciences of Fudan University, West China Institute of Women and Children’s Health, West China Second University Hospital, Sichuan University, Chengdu, China; ^5^ Sichuan Birth Defects Clinical Research Center, West China Second University Hospital, Sichuan University, Chengdu, China; ^6^ Department of Radiotherapy, Cancer Center, State Key Laboratory of Biotherapy, West China Hospital, National Clinical Research Center for Geriatrics, Sichuan University, Chengdu, China; ^7^ Department of Pediatric Cardiology, West China Second University Hospital, Sichuan University, Chengdu, China; ^8^ Department of Pediatric Pulmonology and Immunology, WCSUH-Tianfu·Sichuan Provincial Children’s Hospital, Sichuan University, Meishan, China

**Keywords:** port-wine stains, angiopoietin-2, photodynamic therapy, apoptosis, angiogenesis

## Abstract

Hemoporfin-mediated photodynamic therapy (HMME-PDT) has demonstrated significant advantages in the treatment of Port-wine stains (PWSs). However, the therapeutic efficacy of HMME-PDT remains suboptimal in a subset of patients. Somatic mosaic mutations in *GNAQ* (c.548G>A, p. R183Q) are frequently detected in endothelial cells (ECs) of lesions and represent a common pathogenic mechanism. In this study, we successfully established an *in vitro* model of PWSs by introducing the *GNAQ* p. R183Q mutation into HUVECs using lentiviral infection. Our results revealed that *GNAQ* p. R183Q mutation enhanced ECs proliferation, migration, and angiogenesis. Moreover, the mutation augmented anti-apoptotic mechanisms, thereby conferring heightened resistance to HMME-PDT-induced apoptosis. Residual angiogenic activity persisted following HMME-PDT treatment. These effects are likely mediated by activation of the angiopoietin-2 (ANGPT2)/TIE2/PI3K/AKT signaling axis. Knockdown of ANGPT2 partly reversed these phenotypic alterations and significantly enhanced the efficacy of HMME-PDT. The combination of HMME-PDT with anti-ANGPT2 therapy holds promise for enhancing therapeutic efficacy, suppressing pathological angiogenesis, and ameliorating the clinical manifestations of PWSs.

## 1 Introduction

Port-wine stains (PWSs), also known as nevus flammeus, are congenital capillary malformations (CMs) characterized by dilation and distortion of superficial dermal capillaries, with a reported incidence ranging from 0.1% to 0.8% (Hammill and Boscolo, 2024; Yu et al., 2023; Escobar et al., 2022). Clinically, PWSs initially appear as flat, pale pink macules with well-demarcated margins, commonly affecting the face and neck. Without clinical intervention, these lesions typically persist and may progress with age, becoming darker and thicker, eventually forming nodules or ulcerations (Updyke and Khachemoune, 2017). Beyond cosmetic concerns, approximately 10% of PWSs represent a symptom of Sturge-Weber syndrome (SWS), a complex and severe neurocutaneous disorder (Yeom and Comi, 2022; Boos et al., 2020). SWS is also associated with significant neurological and ocular complications, including seizures, developmental delays, hemiparesis, and glaucoma, profoundly affecting patient quality of life (Dingenen et al., 2024; Solomon and Comi, 2024).

Although the exact pathogenesis remains unclear, recent studies have highlighted the pathogenic role of somatic mutations in the development of PWSs. Among them, a recurrent somatic nonsynonymous single nucleotide variant, *GNAQ* p. R183Q (c.548G>A), is frequently detected in both sporadic and SWS-associated PWSs, with a mutation detection rate approaching 90% and allele frequencies ranging from 1.0% to 18.1% (Shirley et al., 2013; Nguyen et al., 2019; Liu et al., 2022). The *GNAQ* gene encodes the Gαq protein, a G protein α-subunit family member that localizes to the inner surface of the plasma membrane, where it couples with G protein-coupled receptors (GPCRs) to mediate downstream signaling (Sánchez-Fernández et al., 2014; Kimple et al., 2011). The p. R183Q mutation occurs in the Gαq subunit, resulting in impaired guanosine-5′-triphosphate hydrolase (GTPase) activity and constitutive activation of the Gαq protein (Van Trigt et al., 2022; Bichsel and Bischoff, 2019). The sustained activation of Gαq triggers aberrant downstream signaling, including the phosphoinositide 3-kinase/protein kinase B (PI3K/AKT) and phospholipase C-β (PLCβ) pathways, ultimately leading to aberrant endothelial cells (ECs) behavior (Nguyen et al., 2019; Liu et al., 2022; Huang et al., 2022). Notably, this dysregulated signaling cascade promotes the release of Angiopoietin-2 (ANGPT2), a key regulator of vascular remodeling, which in turn facilitates pathological angiogenesis and capillary network expansion, ultimately contributing to the characteristic lesions of PWSs(Huang et al., 2022; Akwii et al., 2019).

In recent years, hemoporfin-mediated photodynamic therapy (HMME-PDT) has emerged as an effective therapeutic option for PWSs. PDT is a photochemical therapy that relies on the combined action of a photosensitizer, specific-wavelength light, and oxygen (Allison and Moghissi, 2013). Following intravenous administration, the photosensitizer selectively accumulates in ECs of abnormal vessels. Upon light activation, it transitions from its ground state to an excited state, transferring energy to molecular oxygen and generating reactive oxygen species (ROS) (Kwiatkowski et al., 2018; Kolarikova et al., 2023), which induce oxidative stress and trigger ECs apoptosis, ultimately leading to the destruction of abnormal blood vessels and clinical improvement in PWS lesions (Kessel and Oleinick, 2018; Kessel, 2019). While pulsed dye laser (PDL) therapy remains the “gold standard” for treating PWSs(Sabeti et al., 2021), HMME-PDT offers several advantages, including high selectivity, minimal invasiveness, and efficacy in PDL-resistant cases, and is now widely used in clinical settings in China (Wang et al., 2024; Zhang et al., 2023; Han et al., 2020). However, substantial therapeutic heterogeneity has been observed in clinical practice. A meta-analysis of 26 studies involving 3,034 patients reported that only 51.5% achieved ≥60% improvement after one to 8.2 treatment sessions, with efficacy strongly influenced by factors such as age, lesion type, and anatomical location (Wang et al., 2023). HMME-PDT tends to be more effective in younger patients, with red and pink lesions responding more favorably than purple ones (Chun-Hua et al., 2021; Zhang et al., 2022). In addition, vascular morphology has been identified as a key determinant of treatment response (Huang et al., 2021). These observations raise the question of whether intrinsic molecular factors, particularly genetic alterations, may underlie differential treatment sensitivity.

To explore this hypothesis, we generated *GNAQ* p. R183Q mutant and wild-type (WT) endothelial cell lines via lentiviral transduction and systematically compared their responses to HMME-PDT. Strikingly, R183Q-mutant cells exhibited marked resistance to HMME-PDT-induced apoptosis, suggesting a survival advantage conferred by aberrant angiopoietin-2 (ANGPT2)/TIE2/PI3K/AKT signaling. Targeting ANGPT2 in combination with HMME-PDT reversed this resistance and significantly enhanced therapeutic efficacy, pointing to a promising combinatorial strategy for improving outcomes in patients with PWSs.

## 2 Materials and methods

### 2.1 Cell lines construction and culture

*GNAQ* p. R183Q mutation, along with green fluorescent protein (GFP) and a puromycin resistance gene, was synthesized by Genewiz (Suzhou, China). Human umbilical vein endothelial cells (HUVECs) were transduced with lentivirus at MOI = 20 using HiTransG P (GeneChem, Cat#REVG005) to enhance efficiency. 48 h post-transduction, positive cells were selected by 2ug/mL puromycin (Biofroxx, Cat#1299MG025) for 7–10 days and were subsequently used in further experiments. The successfully transduced mutant cells were designated as HUVEC-GNAQ-R183Q, while the wild-type control cells were referred to as HUVEC-WT. Transduction efficiency was confirmed by fluorescence microscopy (Olympus, CKX53, Japan) and quantified by flow cytometry (Fortessa, BD Biosciences, USA). *GNAQ* mRNA expression was quantified by qRT-PCR, and Gαq protein levels were analyzed by Western blotting. Both HUVEC-WT and HUVEC-GNAQ-R183Q were cultivated in high-glucose Dulbecco’s modified Eagle’s medium (DMEM, Gibco, Cat#C11995500BT) supplemented with 10% fetal bovine serum (FBS, Gibco, Cat#26140079) and 1% penicillin-streptomycin (Gibco, Cat#15140122) at 37 °C with 5% CO2.

### 2.2 Photosensitizer and device

Hematoporphyrin monomethyl ether (HMME, generic name: hemoporfin), purchased from Shanghai Fudan-Zhangjiang Bio-Pharmaceutical Co., Ltd. (Shanghai, China), served as the photosensitizer. A stock solution of HMME was prepared at 10 mg/mL in 0.9% NaCl and stored in the dark at −20 °C. For experimental use, the stock solution was thawed on ice bed and diluted in DMEM medium to the required working concentration (0–20 μ g/mL). The LED photodynamic therapy apparatus (KN-7200), acquired from Wuhan YaGe Photoelectric Technology Co., Ltd. (Wuhan, China), was employed in this study, emitting light at a precise wavelength of 532 nm.

### 2.3 *In vitro* HMME-PDT treatment

HUVECs were seeded at equal densities into 6-well plates and cultured to 80%–90% confluency over 24 h. The supernatants were then removed, and cells were washed twice with PBS (Gibco, Cat#C14190500BT) before being treated with HMME at varying concentrations and incubated in the dark for 1 h. Prior to laser irradiation, the HMME-containing medium was discarded, and cells were washed three times with PBS. During irradiation, cells in PBS were positioned 10 cm directly beneath the light source for central alignment. A continuous-wave 532 nm laser was applied at energy densities of 3 J/cm^2^ and 4 J/cm^2^. Control groups included untreated cells, cells treated with HMME only, and cells exposed to light only. After irradiation, the medium was replaced with DMEM containing 2% FBS to minimize proliferation rate differences between cell lines, and cells were incubated in the dark for an additional 24–72 h. Morphological changes under different conditions and time points were observed and documented using an inverted microscope.

### 2.4 Cell counting kit-8 (CCK-8) assay

The proliferation rates of WT and R183Q cells were assessed using a CCK-8 assay (Dojindo, Cat#CK04) according to the manufacturer’s guidelines. Cells were seeded at a density of 5 × 10^3^ cells per well in 100 µL of complete DMEM in 96-well plates. At designated time points (0, 12, 24, 48, and 72 h), 10 µL of CCK-8 solution was added to each well, and plates were incubated at 37 °C for 1 h. Absorbance at 450 nm was measured using a Biotek Synergy H1 microplate reader (Biotek, USA). The relative absorbance values were used to quantify the proliferation rates of both cells over time.

To evaluate cell viability following HMME-PDT treatment and determine the half-maximal inhibitory concentration (IC_50_), HUVECs were seeded at an equal density of 1 × 10^4^ cells per well in 96-well plates. Cells were treated with varying concentrations of HMME (0, 2.5, 5, 7.5, 10, 15, and 20 μ g/mL) and then subjected to PDT as above described. Following treatment, cells were maintained in the dark for 24 and 72 h. At each time point, 10 µL of CCK-8 solution was added to each well, followed by incubation for 1 h. Absorbance at 450 nm was measured on the same Biotek Synergy H1 microplate reader. Cell viability was calculated relative to untreated controls using the formula:

$$\text{Cell viability }\left(\%\right)=\frac{\text{OD}\;\left(\text{experiment}\right)-\text{OD}\;\left(\text{blank}\right)}{\text{OD}\left(\text{control}\right)-\text{OD}\;\left(\text{blank}\right)}\times 100\%$$

For IC_50_ determination, dose-response curves were generated using GraphPad Prism v.10.0 to calculate the IC_50_ values, representing the concentration of HMME required to achieve a 50% reduction in cell viability.

### 2.5 Apoptosis detection via flow cytometry

After overnight serum starvation in 6-well plates, HUVECs were treated with HMME-PDT as previously described. 24 or 72 h post-PDT, cells were collected for apoptosis analysis using the Annexin V, 633 Apoptosis Detection Kit (Dojindo, Cat#AD11). Following the manufacturer’s protocol, 5 µL of Annexin V, 633 conjugate and 5 µL of PI Solution were added to each sample, gently mixed, and incubated at room temperature in the dark for 15 min. Labeled cells were resuspended in binding buffer and analyzed using a flow cytometer (Fortessa, BD Biosciences, USA) to assess the proportion of apoptotic cells. Experiments were performed independently in triplicate, and data were analyzed with FlowJo v.10.8.1.

### 2.6 Detection of mitochondrial membrane potential (Δψm)

Δψm was assessed using a tetramethylrhodamine ethyl ester (TMRE)-based assay kit (Beyotime, C2001S) according to the manufacturer’s instructions. After treatment, cells were collected and incubated with TMRE (1 μL per 1 × 10^6^ cells) at 37 °C for 30 min in the dark. Subsequently, cells were washed with PBS and immediately analyzed by flow cytometry using a Fortessa instrument (BD Biosciences, USA). Data were analyzed using FlowJo (v10.8.1).

### 2.7 Capillary network formation on a matrigel matrix

Capillary network formation was assessed in both WT and R183Q cells before and 24 h post-PDT. HUVECs (1.5 × 10^5^ cells) were plated in 24-well plates pre-coated with 30 µL Matrigel (Corning, Cat#356231) per well. Cells were incubated in 500 µL of complete DMEM for 4–6 h to allow capillary network formation. Bright-field images were captured using an Olympus CKX53 microscope (Japan). Quantitative analysis was conducted using ImageJ software to measure junctions, nodes, and the cumulative structure length.

### 2.8 Scratch wound migration assay

HUVECs were plated in 6-well plates, and linear scratches were made using a sterile 200 µL pipette tip. After scratching, cells were cultured in 2% FBS and monitored under a microscope in bright-field at 0, 24 and 48 h to assess wound closure.

### 2.9 Transwell migration analysis

8 µm-pore Transwell inserts (Corning, Cat#3422) were positioned in 24-well plates. HUVECs were resuspended in serum-free medium and seeded into the upper chamber of the insert at a density of 5 × 10^4^ cells per well, while the lower chamber was supplemented with medium containing 10% FBS to serve as a chemoattractant. After a 24-h incubation, non-migrated cells on the upper surface of the membrane were carefully removed with a cotton swab, and cells that had migrated to the underside were fixed in 4% paraformaldehyde and stained with 0.1% crystal violet. This experiment was performed in triplicate, with five random fields per well imaged using an Olympus CKX53 microscope (Japan) for quantitative analysis. Migrated cells were counted using ImageJ software.

### 2.10 Western blotting

Total protein was extracted from HUVECs using RIPA lysis buffer supplemented with protease inhibitor cocktail (Roche, Cat#4693159001) and phosphatase inhibitor cocktails (Roche, Cat#04906837001) to prevent protein degradation. Cell lysates were centrifuged at 12,000 r/min for 15 min at 4 °C, and the supernatant was promptly collected. Protein concentrations were determined using a BCA Protein Assay Kit (Beyotime, Cat#P0012S). Equal amounts of protein were then mixed with 4× Laemmli loading buffer (Bio-Rad, Cat#1610747) and denatured by heating at 95 °C for 5 min.

Prepared protein samples were separated on 6%–12% SDS-PAGE gels and subsequently transferred onto PVDF membranes (Millipore, USA) by a wet transfer apparatus (Bio-Rad, USA). Following transfer, membranes were blocked with 5% non-fat milk in TBST for 2 h at room temperature to reduce nonspecific binding. Membranes were incubated overnight at 4 °C with primary antibodies against target proteins (anti-β-actin antibody, 1:50,000, Proteintech, Cat#66009-1-Ig; anti-Gαq antibody, 1:500, Huabio, Cat#HA721328; anti-AKT antibody, 1:1000, CST, Cat#4691S; anti-Phospho-AKT (Ser473) antibody, 1:2000, CST, Cat#4060S; anti-pro-caspase-3 antibody, 1:2000, Huabio, Cat#ET1602-39; anti-cleaved-caspase-3 antibody, 1:1000, CST, Cat#9662S; anti-Bax antibody, 1:20,000, Huabio, Cat#ET1603-34; anti-Bcl-2 antibody, 1:5000, Huabio, Cat#ET1603-11; anti-PI3K p110α antibody, 1:1000, CST, Cat#4249S; anti-TIE2 antibody, 1:1000, Huabio, Cat#HA722732; anti-Phospho-TIE2 (Tyr992) antibody, 1:1000, Affinity, Cat#AF2424). After washing, membranes were incubated with HRP-conjugated secondary antibodies (Goat anti-Rabbit, 1:5000, Millipore, Cat#AP132P; Goat anti-Mouse, 1:5000, Millipore, Cat#AP124P) for 1 h at room temperature. Protein bands were visualized using an enhanced chemiluminescence (ECL) detection kit (Biosharp, Cat#BL520A) and imaged with a G:BOX Chemi XRQ imaging system (Syngene, UK). Band intensities were quantified using ImageJ software, with β-Actin used as the internal control for normalization of protein expression levels.

### 2.11 Quantitative real-time PCR (qRT‒PCR)

Total RNA was extracted from approximately 1 × 10^6^ cells using the RNAprep Pure Micro Kit (TIANGEN, Cat#DP420) according to the manufacturer’s instructions. RNA concentration and purity were assessed using a NanoDrop spectrophotometer (Thermo Fisher Scientific, USA). cDNA synthesis was performed using the Transcriptor First Strand cDNA Synthesis Kit (Roche, Cat#04897030001). qRT-PCR was then carried out on a LightCycler 96 Real-Time PCR System (Roche, Switzerland) using GoTaq® qPCR Master Mix (Promega, Cat#A6002), with each reaction carried out in a 10 µL volume according to the manufacturer’s protocol. Relative gene expression levels were calculated as fold changes normalized to *GAPDH* using the 2^−ΔΔCt^ method. Primers for the target genes were synthesized by Sangon Biotech (Shanghai) Co., Ltd., with sequences provided in Supplementary Table S1.

### 2.12 ELISA

Before and 24 h post-PDT treatment, supernatants from HUVECs cultures were collected for analysis. The levels of ANGPT2 were quantified using enzyme-linked immunosorbent assay (ELISA) kits (Eabscience, cat#E-EL-H0008) following the manufacturer’s protocol.

### 2.13 siRNA-mediated gene knockdown

HUVECs were transfected with three specific siRNAs targeting ANGPT2 (si-ANGPT2#1, #2, and #3) or a negative control siRNA (si-NC), all synthesized by Tsingke Biotech Co., Ltd. (Beijing, China). Cells were seeded in 6-well plates and cultured to 60%–70% confluency prior to transfection. Transfections were carried out using Lipofectamine 3000 (Thermo Fisher Scientific, Cat#L3000015) with a final siRNA concentration of 50 nM, according to the manufacturer’s protocol. After 48 h of transfection, ELISA or qRT‒PCR was performed to test knockdown efficiency. Detailed sequences information on the siRNAs used was provided in Supplementary Table S2.

### 2.14 Bulk RNA-seq

We downloaded the bulk RNA-seq dataset GSE186998 from the GEO database (https://www.ncbi.nlm.nih.gov/geo/) (Huang et al., 2022). This dataset comprises Lentiviral-engineered HUVECs harboring the *GNAQ* p. R183Q mutation and corresponding WT controls, with three biological replicates per condition. Quality assessment of the raw FASTQ files was performed using FastQC (v.0.12.1) to ensure data integrity and reliability. Cleaned reads were then aligned to the human reference genome (hg38) using HISAT2 (v.2.2.1), and gene-level quantification was conducted with FeatureCounts (v.2.0.6). After normalization, differentially expressed genes (DEGs) analysis between R183Q and WT groups was performed using the DESeq2 R package, with DEGs defined by a false discovery rate (FDR) threshold of <0.10. To further analysis, gene set enrichment analysis (GSEA)-based Gene Ontology (GO) enrichment and Kyoto Encyclopedia of Genes and Genomes (KEGG) enrichment analysis were conducted using the clusterProfiler R package. Gene sets achieving a *p*-value <0.05 and an FDR <0.25 were deemed significantly enriched.

### 2.15 Statistical analysis

All statistical analyses were performed using the R software v.4.4.1 or GraphPad Prism v.10.0, with results expressed as mean ± SD. For two-group comparisons, an unpaired or paired two-tailed Student’s t-test was used for equal variances, and Welch’s t-test for unequal variances. The Mann-Whitney U test was applied for non-normally distributed data. For comparisons involving three or more groups, one-way or two-way analysis of variance (ANOVA) was used for normally distributed data, followed by Dunnett’s test for comparisons to a control group or Sidak’s test for multiple pairwise comparisons. *P* < 0.05 was considered statistically significant.

## 3 Results

### 3.1 Lentiviral-mediated construction and validation of HUVEC-GNAQ-R183Q cells

**FIGURE 1:**
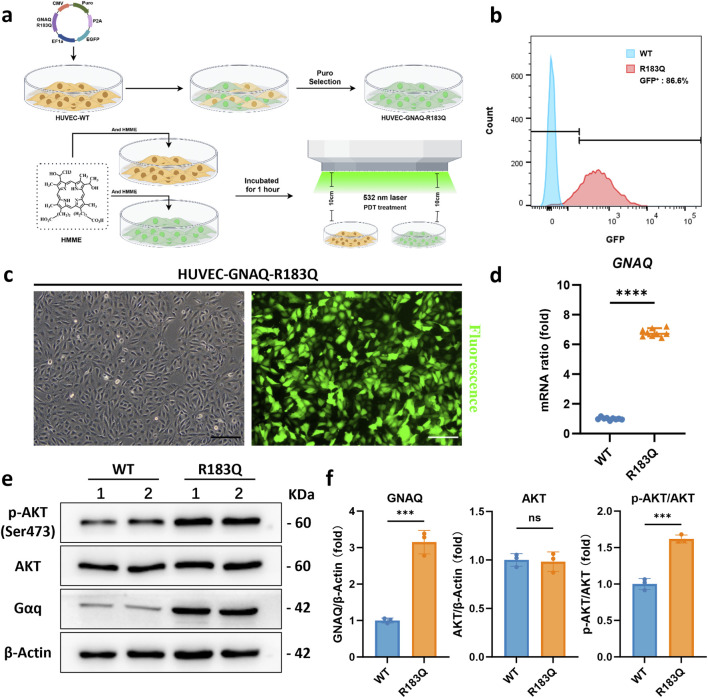
Lentiviral-Mediated Construction and Validation of HUVEC-GNAQ-R183Q Cells. **(a)**, Schematic of lentiviral transduction, GFP-positive cell selection, and the establishment of HMME-PDT system. **(b)**, Flow cytometry analysis showing that 84.8% of R183Q cells were GFP-positive (red), compared with the GFP-negative WT cells (blue). **(c)**, Representative bright-field and GFP fluorescence images of R183Q cells. Scale bars: 200 μm **(d)**, qRT-PCR analysis of *GNAQ* mRNA expression in WT and R183Q cells, normalized to *GAPDH*. (n = 3, mean ± s.d.); ****P < 0.0001. **(e,f)**, Western blotting analysis of Gαq, total AKT, and p-AKT (Ser473) protein levels in WT and R183Q cells. β-Actin was used as a loading control. (n = 3, mean ± s.d.); ***P < 0.001, ns: no significance.

In order to more accurately simulate the disease state of PWSs, we engineered a lentivirus carrying the *GNAQ* p. R183Q mutation and successfully established a stable HUVEC-GNAQ-R183Q cell line via lentiviral infection (Figure 1a), with the mutant sequence detailed in Supplementary Material 1. Flow cytometry and fluorescence microscopy were performed to confirm efficient integration of the R183Q mutation in ECs. Flow cytometry analysis indicated that 86.6% of the infected cells expressed GFP, sharply distinguishing them from GFP-negative WT controls (Figure 1b). Fluorescence microscopy further confirmed these results, showing robust and uniform GFP expression across the R183Q cells (Figure 1c). We also assessed RNA and protein expression changes following lentiviral infection. As expected, R183Q cells exhibited significantly increased *GNAQ* mRNA levels (Figure 1d) and elevated Gαq protein expression (Figures 1e,f), confirming the successful construction of the R183Q cell line. Notably, while AKT phosphorylation was detected in both R183Q and WT cells, the activation level was significantly higher in the R183Q cells, consistent with previous studies implicating AKT as a downstream target of Gαq point mutations. Collectively, these results validate the HUVEC-GNAQ-R183Q cell line as a reliable model for studying the mutation’s effects in ECs.

### 3.2 Effects of R183Q mutation on the biological properties of ECs

**FIGURE 2:**
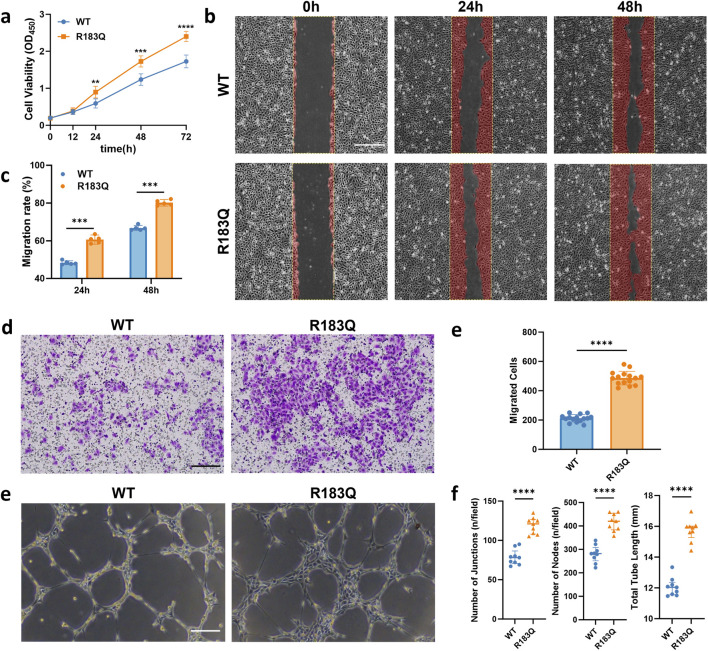
Effects of R183Q Mutation on the Biological Properties of ECs. **(a)**, Proliferation rates of WT and R183Q cells assessed by CCK-8 assay at multiple time points (0, 12, 24, 48, and 72 h). (n = 5, mean ± s.d.); ****P < 0.0001, ***P < 0.001, **P < 0.01. **(b,c)**, Representative images of WT and R183Q cells at specified time points in the scratch assay, with the red area indicating the migrated region (n = 5, scale bars: 200 μm), and quantitative analysis of cell migration at 24 and 48 h (mean ± s.d.); ***P < 0.001. **(d,e)**, Representative Transwell images showing crystal violet-stained migrated WT and R183Q cells (n = 3, scale bars: 200 μm) and quantification of migrated cells per field. (mean ± s.d.); ****P < 0.0001. **(e,f)**, Representative images showing capillary network formation by WT and R183Q cells on Matrigel. (n = 3, scale bars: 200 μm) and quantification analysis of network parameters. (mean ± s.d.); ****P < 0.0001.

To investigate the impact of the R183Q mutation on the biological characteristics of ECs, we conducted a series of functional assays. First, cell proliferation was evaluated using a CCK-8 assay, revealing that the proliferation rate of R183Q cells significantly exceeded that of WT (Figure 2a). Next, we assessed cell migration through both scratch and Transwell assays. In the scratch assay, R183Q cells demonstrated a faster wound closure compared to WT, indicating enhanced migratory capability (Figures 2b,c). Similarly, in the Transwell migration assay, R183Q cells displayed a substantially higher migration across the membrane than WT (Figures 2d,e). Finally, tube formation ability was assessed by seeding R183Q and WT ECs on Matrigel, allowing capillary-like network formation over a 6-h incubation period. Compared to WT, R183Q cells formed a more extensive network with increased junctions and nodes, as well as greater total network length (Figures 2f,g). Together, these findings suggest that the *GNAQ* p. R183Q mutation significantly enhances proliferation, migration, and angiogenic potential in ECs, which may contribute to the pathogenesis of PWSs associated with this mutation.

### 3.3 ECs with R183Q Mutation Exhibit Negative Regulation of apoptotic signaling Enhanced Resistance to HMME-PDT

**FIGURE 3:**
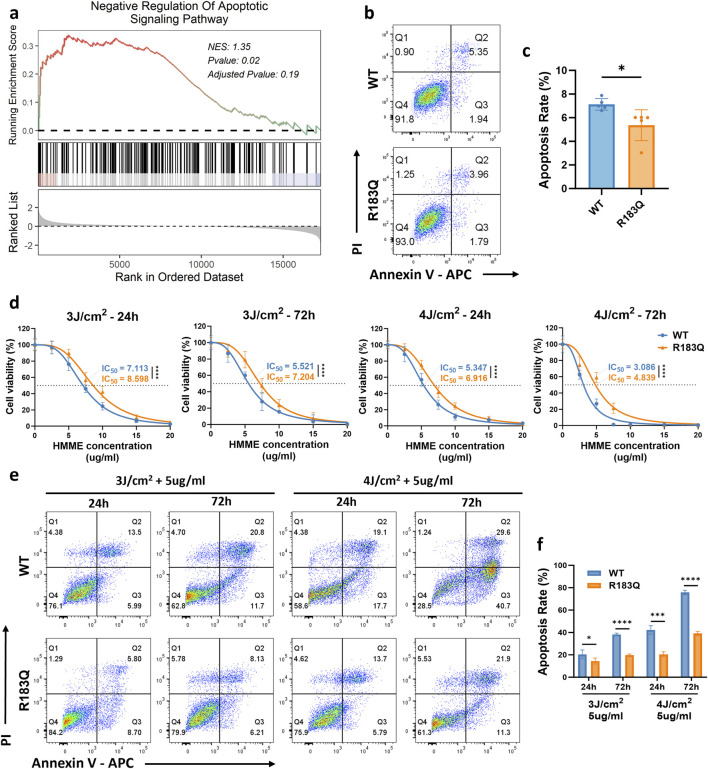
ECs with R183Q Mutation Exhibit Negative Regulation of Apoptotic Signaling and Enhanced Resistance to HMME-PDT. **(a)**, GSEA plot indicating significant enrichment of the negative regulation of the apoptotic signaling pathway in R183Q mutant cells. NES, normalized enrichment score. **(b,c)**, Annexin V/PI flow cytometric analysis comparing apoptotic rates in WT and R183Q cells. (n = 3, mean ± s.d.); *P < 0.05. **(d)**, WT and R183Q cells were treated with HMME-PDT across different conditions (HMME 0–20 μg/mL; Energy densities three or 4 J/cm^2^). Cell viability at 24 and 72 h post-PDT was evaluated using the CCK-8 assay, and dose–response curves illustrate the IC_50_ (dashed lines). (n = 3, mean ± s.d.); ****P < 0.0001. **(e,f)**, Annexin V/PI flow cytometric comparison of apoptotic responses in WT and R183Q cells following HMME-PDT (5 μg/mL) at energy densities of three or 4 J/cm^2^, evaluated at 24 and 72 h post-PDT. (n = 3, mean ± s.d.); *P < 0.05, ***P < 0.001, ****P < 0.0001.

GSEA analysis revealed a significant enrichment of the Negative Regulation of Apoptotic Signaling Pathway in R183Q cells (*P* = 0.02) (Figure 3a), suggesting a potential suppression of apoptosis. Consistently, Annexin V/PI flow cytometric analysis further confirmed this finding, demonstrating a reduced apoptotic rate in R183Q cells compared to WT cells (Figures 3b,c).

To investigate the differential responses of WT and R183Q mutant ECs to treatment, a HMME-PDT system was established in accordance with the protocol detailed in the Methods section (Figure 1a). Both cell types were exposed to light intensities of 0 J/cm^2^, 3 J/cm^2^, or 4 J/cm^2^ and treated with HMME at concentrations ranging from 0 to 20 μg/mL. Cellular responses were assessed at 24 and 72 h post-PDT to evaluate both immediate and delayed effects. Initially, the cell viability of WT and R183Q cells following HMME-PDT treatment was quantitatively assessed using the CCK-8 assay. Dose-response curves were generated for both cell types (Figure 3d). Within a certain range, both WT and R183Q cells exhibited a significant decrease in viability with increasing HMME concentration and/or light intensity. However, IC_50_ calculations consistently showed that R183Q cells had higher IC_50_ values than WT cells, indicating greater resistance to HMME-PDT-induced cytotoxicity. Additionally, the decline in cell viability was more pronounced at 72 h than at 24 h in both cell types, suggesting a time-dependent cumulative effect of HMME-PDT treatment. Following treatment, morphological changes in ECs were examined using an inverted microscope (Supplementary Figure S1). In the control groups (untreated, HMME only, and LED light only), cells retained their normal adherent morphology without noticeable alterations at both 24 and 72 h. In contrast, HMME-PDT treatment induced significant morphological changes in both WT and R183Q cells, including cell rounding, shrinkage, detachment, and the presence of floating debris. Among adherent cells, increased pseudopodia formation and vacuolation were observed, indicative of cellular stress. Notably, these morphological alterations were more pronounced at 72 h than at 24 h. Furthermore, WT cells exhibited more extensive changes compared to R183Q mutant cells, suggesting a differential sensitivity to HMME-PDT between the two cell types.

Previous studies have shown that PDT exerts its cytotoxic effects primarily through the induction of apoptosis (Mroz et al., 2011). Therefore, Annexin V/PI flow cytometry was performed to quantify apoptosis in R183Q and WT cells under various treatment conditions. Flow cytometry analysis revealed that WT and R183Q cells in the untreated, HMME-only, and LED-only control groups exhibited minimal apoptosis, with total apoptosis rates generally remaining below 10% (Supplementary Figure S2). However, in the HMME-PDT treatment group, apoptosis rates—including early apoptosis (FITC-positive, PI-negative), late apoptosis (FITC-positive, PI-positive), and total apoptosis—significantly increased in WT cells, particularly at 72 h post-PDT (Figures 3d,e). Although R183Q cells also showed increased apoptosis under the same conditions, their apoptosis rates were significantly lower than those observed in WT cells (Figures 3d,e). Specifically, at 24 h post-PDT with 5 μg/mL HMME and 4 J/cm^2^ light intensity, the total apoptosis rate in WT cells reached approximately 40%, whereas R183Q cells exhibited a total apoptosis rate of only around 20%. By 72 h, the total apoptosis rate in WT cells further increased to approximately 75%, while in R183Q cells, it reached around 40%, indicating a statistically significant difference between the 2 cell types. Collectively, these findings confirm a markedly attenuated apoptotic response in R183Q mutant ECs compared to WT cells following HMME-PDT treatment.

### 3.4 R183Q mutation ECs Inhibit activation of the mitochondrial apoptotic pathway via TIE2/PI3K/AKT signaling

To better simulate clinical conditions and obtain representative data on HMME-PDT responses, we combined CCK-8 cell viability assays with flow cytometry apoptosis analysis identify conditions that resulted in moderate apoptosis rates (5 μg/mL HMME, 3 J/cm^2^ light intensity, assessed at 24 and 72 h post-PDT) as representative for subsequent HMME-PDT experiments. Based on these optimized parameters, we will further investigate the potential mechanisms underlying the resistance to apoptosis in R183Q mutant cells under treatment.

**FIGURE 4:**
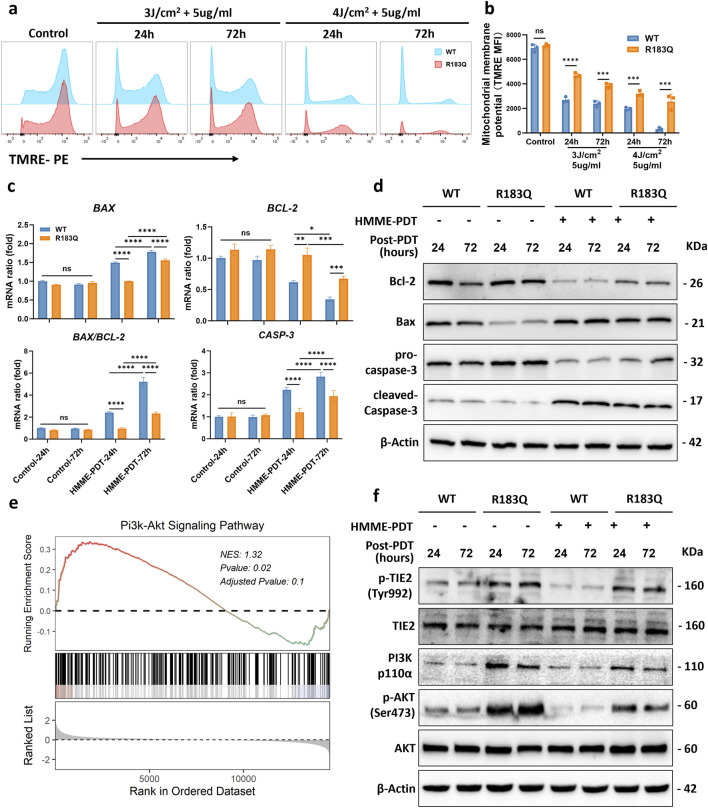
R183Q Mutation ECs Inhibit Activation of the Mitochondrial Apoptotic Pathway via TIE2/PI3K/AKT Signaling. **(a)**, Mitochondrial membrane potential was assessed in WT and R183Q cells by TMRE staining following HMME-PDT treatment (5 μg/mL HMME, three or 4 J/cm^2^), with analysis at 24 and 72 h post-PDT. Flow cytometry histograms depict TMRE fluorescence intensity in control and treated groups. **(b)**, Quantification of mitochondrial membrane potential based on mean fluorescence intensity (MFI) of TMRE staining (n = 3; mean ± s.d.). ****P < 0.0001, ***P < 0.001, ns: not significant. **(c)**, qRT-PCR was performed to determine *BAX*, *BCL-2*, and *CASP3* mRNA levels in WT and R183Q cells pre- and post-PDT, with *GAPDH* as an internal control. Cells were exposed to either control conditions (0 μg/mL HMME, 0 J/cm^2^) or HMME-PDT conditions (5 μg/mL HMME, 3 J/cm^2^) and harvested at 24 and 72 h post-PDT. (n = 3, mean ± s.d.); ****P < 0.0001, ***P < 0.001, **P < 0.01, *P < 0.05, ns: no significance. **(d)**, Western blotting analysis of Bcl-2, Bax, pro-caspase-3, and cleaved-caspase-3 expression in WT and R183Q cells before and after HMME-PDT treatment. β-Actin served as the loading control. (n = 3) **(e)**, GSEA plot indicating significant enrichment of the PI3K/AKT signaling pathway in R183Q mutant cells. **(f)**, Western blotting analysis of total TIE2, p-TIE2 (Tyr992), PI3K (p110α), total AKT, and p-AKT (Ser473) levels in WT and R183Q cells before and after HMME-PDT treatment. β-Actin served as the loading control. (n = 3).

Apoptosis is primarily activated through two pathways: mitochondrial-dependent and mitochondrial-independent. Previous studies have shown that PDT-induced apoptosis predominantly follows the mitochondrial-dependent pathway (Kessel, 2020). To assess changes in Δψm, cells were stained with TMRE and analyzed by flow cytometry using the PE channel (Figures 4a,b). Under control condition, both WT and R183Q cells exhibited similarly high Δψm, with mean fluorescence intensity (MFI) values around 7000. Following HMME-PDT treatment, a reduction in MFI was observed in both cell types, indicating mitochondrial depolarization. Consistent with the apoptosis assay, the decline in Δψm was more pronounced in WT cells compared to R183Q cells under the same HMME concentration and light energy densities conditions. To further investigate the molecular basis of this difference, we analyzed the expression of key regulators involved in the mitochondrial apoptotic pathway in both cell types, with or without HMME-PDT treatment. At the RNA level, the results showed that under control conditions, there were no significant differences in the expression levels of *BAX*, *BCL-2*, and *CASP3* between R183Q and WT cells (Figure 4c). However, after HMME-PDT treatment for 24 and 72 h, the *BAX/BCL-2* ratio and *CASP3* expression significantly increased in both cell types, indicating activation of the mitochondrial-dependent apoptotic pathway (Figure 4c). Consistently, the upregulation of the *BAX/BCL-2* ratio and *CASP3* in R183Q cells was less pronounced than in WT cells. Similar trends were observed at the protein level. After HMME-PDT treatment, the anti-apoptotic protein Bcl-2 was downregulated, while the pro-apoptotic protein Bax was upregulated, resulting in an increased Bax/Bcl-2 ratio. However, this increase was less pronounced in R183Q cells than in WT cells (Figure 4d). Additionally, both R183Q and WT cells exhibited pro-caspase-3 cleavage into its active form, cleaved-caspase-3, which was associated with a reduction in pro-caspase-3 levels and a concurrent increase in cleaved-caspase-3 expression. This effect was more pronounced in WT cells, whereas the activation of cleaved-caspase-3 was significantly attenuated in R183Q mutant cells (Figure 4d). Together, these findings provide strong evidence that R183Q mutant cells exhibit resistance to HMME-PDT-induced activation of the mitochondrial apoptotic pathway.

GSEA analysis identified a significant enrichment of the PI3K-AKT signaling pathway in R183Q cells (*P* = 0.02) (Figure 4e), suggesting its potential involvement in mediating resistance to HMME-PDT-induced apoptosis. To further validate this finding, Western blot analysis was performed to assess the expression levels of key PI3K-AKT pathway components. The results revealed that, in the absence of treatment, R183Q cells exhibited significantly higher levels of PI3K and p-AKT/AKT compared to WT cells, and this difference persisted after HMME-PDT treatment (Figure 4f). Furthermore, to investigate upstream regulatory mechanisms, the expression of total Tyrosine Kinase with Immunoglobulin-like and EGF-like Domains 2 (TIE2) and phosphorylated TIE2 (p-TIE2) was examined. Consistently, R183Q cells displayed elevated p-TIE2/TIE2 levels relative to WT cells, both before and after HMME-PDT treatment, indicating potential activation of the TIE2/PI3K/AKT signaling axis.

### 3.5 ANGPT2 acts as a key mediator driving HMME-PDT resistance and angiogenesis in R183Q cells

**FIGURE 5:**
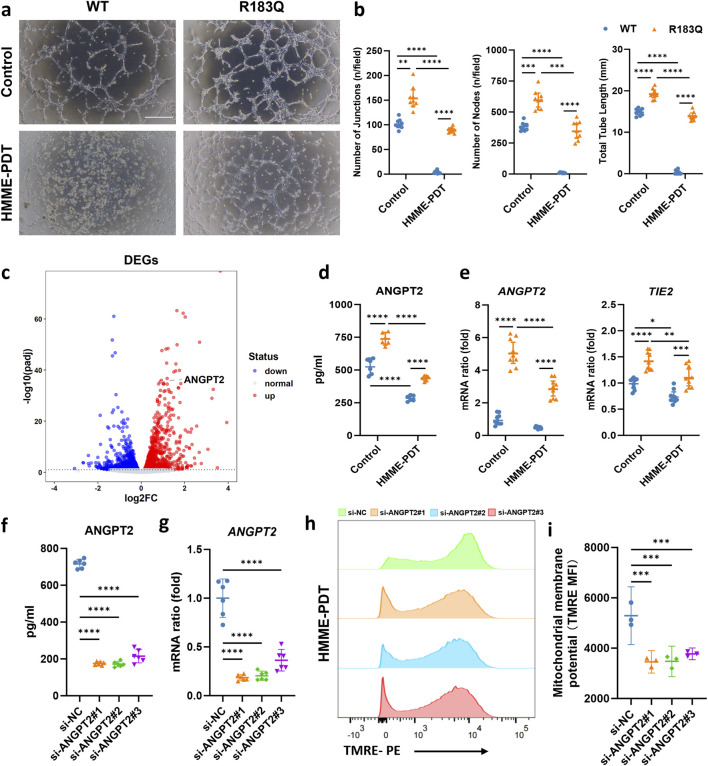
ANGPT2 Acts as a Key Mediator Driving HMME-PDT Resistance and Angiogenesis in R183Q Cells. **(a,b)**, Representative bright-field images showing capillary network formation by WT and R183Q cells on Matrigel, before and after HMME-PDT treatment. (n = 3, scale bars: 200 μm) and quantification analysis of network parameters. (mean ± s.d.); ****P < 0.0001, ***P < 0.001, **P < 0.01. **(c)**, Volcano plot displaying DEGs in R183Q *versus* WT cells, highlighting *ANGPT2*. DEGs, differentially expressed genes. **(d)**, Quantification of ANGPT2 levels in the supernatants of WT and R183Q cells before and after HMME-PDT treatment, measured by ELISA. (n = 3, mean ± s.d.); ****P < 0.0001. **(e)**, qRT-PCR analysis of *ANGPT2* and *TIE2* mRNA expression in WT and R183Q cells before and after HMME-PDT treatment, normalized to *GAPDH*. (n = 3, mean ± s.d.); ****P < 0.0001, **P < 0.001, **P < 0.01. **(f)**, siRNA-mediated knockdown of *ANGPT2* in R183Q cells, followed by quantification of ANGPT2 levels in cell supernatants via ELISA. (n = 3, mean ± s.d.); ****P < 0.0001. **(g)**, qRT-PCR validation of *ANGPT2* mRNA levels in R183Q cells following siRNA knockdown. (n = 3, mean ± s.d.); ****P < 0.0001. **(h)**, Flow cytometry histograms of TMRE fluorescence intensity in R183Q cells transfected with si-NC or si-ANGPT2 (#1–3), 24 h after HMME-PDT treatment (5 μg/mL HMME, 3 J/cm^2^). **(i)**, Quantification of mitochondrial membrane potential based on MFI of TMRE staining (n = 3; mean ± s.d.). ***P < 0.001.

To assess the angiogenic capacity of WT and R183Q mutant cells, we performed a capillary network formation assay on a Matrigel matrix under control and HMME-PDT-treated conditions. Under control conditions, R183Q cells exhibited significantly enhanced capillary formation compared to WT cells after 6 h of incubation, aligning with previous findings (Figures 5a,b). At 24 h post-PDT, capillary formation was severely impaired in WT cells, whereas R183Q cells retained a markedly greater capacity to form capillary-like structures (Figures 5a,b).

To explore the molecular mechanisms underlying HMME-PDT resistance and enhanced angiogenesis in R183Q cells, we performed a differential gene expression analysis between WT and R183Q cells. Among the identified DEGs, *ANGPT2* was significantly upregulated in R183Q cells compared to WT cells, suggesting its potential role as a key regulator of these phenotypic changes (Figure 5c). To further validate this finding at the protein level, we measured ANGPT2 secretion in WT and R183Q cells before and after HMME-PDT treatment using ELISA. The results confirmed that ANGPT2 secretion was consistently higher in R183Q cells compared to WT cells under both control and HMME-PDT-treated conditions (Figure 5d). At the RNA level, qRT-PCR analysis showed that although HMME-PDT treatment reduced *ANGPT2* expression, R183Q cells still exhibited significantly higher *ANGPT2* mRNA levels than WT cells in both conditions (Figure 5e). Similarly, the expression pattern of *TIE2*, the receptor of *ANGPT2*, mirrored this trend, with R183Q cells consistently showing elevated TIE2 mRNA levels compared to WT cells in both control and HMME-PDT-treated conditions, despite a general reduction following HMME-PDT treatment (Figure 5e). These findings suggest that *ANGPT2* functions as a key mediator driving both HMME-PDT resistance and angiogenesis in R183Q cells.

### 3.6 Knockdown of ANGPT2 attenuates HMME-PDT resistance and mitigates pathological angiogenesis in R183Q cells

Next, we explored the feasibility of combining anti-ANGPT2 therapy with HMME-PDT treatment in R183Q cells by using siRNA to knock down the *ANGPT2* gene. ELISA results confirmed the knockdown efficiency, showing that si-ANGPT2#1, #2, and #3 reduced *ANGPT2* secretion by more than 70% (Figure 5f). Similar results were observed at the RNA level, with a significant reduction in *ANGPT2* expression (Figure 5g). After 48 h of *ANGPT2* knockdown in R183Q cells using siRNA, cells were subjected to control and HMME-PDT treatment condition (5 μg/mL HMME, 3 J/cm^2^ light intensity). Bright-field microscopy was used to examine the morphological changes of R183Q cells after treatment. The combination of anti-ANGPT2 therapy with HMME-PDT led to more pronounced morphological alterations (Supplementary Figure S3). Specifically, cells displayed a significant increase in shrinkage and a markedly higher number of floating, detached cells, suggesting greater cytotoxicity. Among the remaining adherent cells, pseudopodia formation was more prominent, and cytoplasmic vacuolization was also notably enhanced.

**FIGURE 6:**
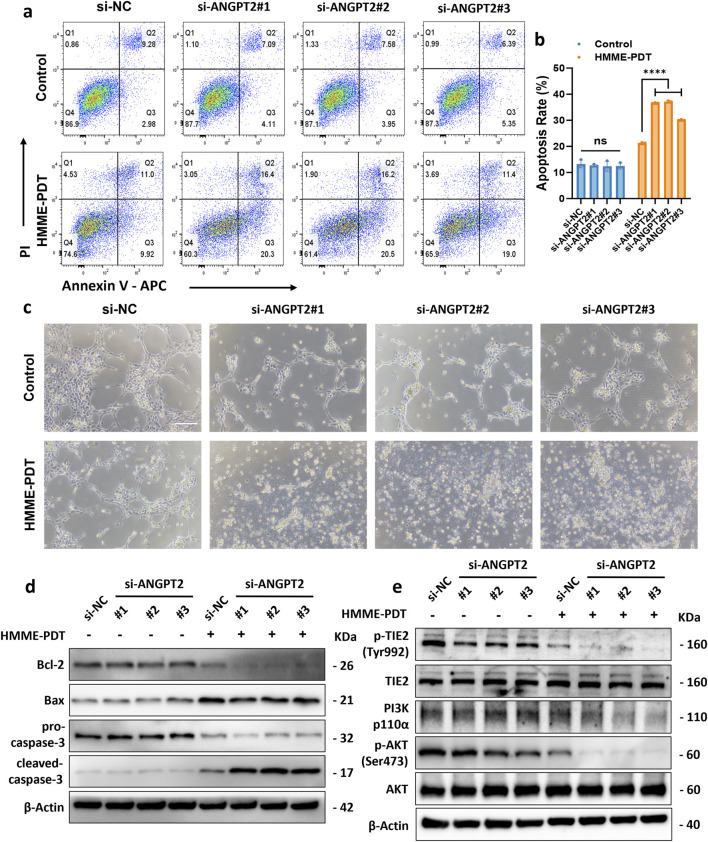
Knockdown of *ANGPT2* attenuates HMME-PDT resistance and mitigates pathological angiogenesis in R183Q cells. **(a,b)**, Annexin V/PI flow cytometric analysis of apoptosis in R183Q cells transfected with si-NC or si-ANGPT2 (#1–3). Cells were exposed to either control conditions (0 μg/mL HMME, 0 J/cm^2^) or HMME-PDT treatment (5 μg/mL HMME, 3 J/cm^2^) and analyzed 24 h post-PDT. (n = 3, mean ± s.d.); ****P < 0.0001, ns: no significance. **(c)**, Representative bright-field images depicting capillary network formation by R183Q cells transfected with si-NC or si-ANGPT2 (#1–3) on Matrigel, before and after HMME-PDT exposure. (n = 3, scale bars: 200 μm). **(d)**, Western blotting analysis of Bcl-2, Bax, pro-caspase-3, and cleaved-caspase-3 expression in R183Q cells transfected with si-NC or si-ANGPT2 (#1–3), before and after HMME-PDT treatment. β-Actin was used as a loading control. (n = 3) **(e)**, Western blotting analysis of total TIE2, p-TIE2 (Tyr992), PI3K (p110α), total AKT, and p-AKT (Ser473) expression levels in R183Q cells transfected with si-NC or si-ANGPT2 (#1–3), before and after HMME-PDT treatment. β-Actin served as the loading control. (n = 3).

Δψm was assessed in R183Q cells transfected with si-NC or si-ANGPT2 (#1, #2, and #3) using TMRE staining followed by flow cytometric analysis (Figures 5h,i). After HMME-PDT treatment, cells transfected with si-NC exhibited a mean TMRE MFI of 5290 ± 462. In contrast, cells transfected with si-ANGPT2#1, #2, and #3 showed a further reduction in Δψm, with MFI values of 3456 ± 179, 3479 ± 243, and 3773 ± 93, respectively (*P* < 0.001), indicating that ANGPT2 knockdown enhanced mitochondrial depolarization following HMME-PDT. Apoptosis was then assessed using Annexin V/PI flow cytometry under both conditions. The results demonstrated that HMME-PDT combined with si-ANGPT2 treatment significantly increased apoptosis in R183Q cells, raising the apoptotic rate from approximately 25% to nearly 40%, thereby markedly enhancing the sensitivity of R183Q cells to HMME-PDT (Figures 6a,b). To further evaluate the impact on angiogenesis, a capillary network formation assay was performed on a Matrigel matrix using si-NC and si-ANGPT2#1, #2, and #3 under both control and HMME-PDT-treated conditions. *ANGPT2* knockdown alone significantly reduced the angiogenic capacity of R183Q cells. Notably, when combined with HMME-PDT, pathological capillary formation was almost completely abolished in all si-ANGPT2 groups, with a more pronounced therapeutic effect observed (Figure 6c).

To further elucidate the molecular mechanisms through which *ANGPT2* knockdown enhances HMME-PDT sensitivity and suppresses pathological angiogenesis in R183Q cells, Western blotting analysis was conducted. The results revealed that si-ANGPT2 combined with HMME-PDT treatment significantly reduced the expression of the anti-apoptotic protein Bcl-2, while Bax levels nearly remained unchanged, leading to a marked decrease in the Bax/Bcl-2 ratio. Additionally, the cleavage of pro-caspase-3 into its active form, cleaved-caspase-3, was significantly increased, indicating robust activation of the mitochondrial apoptotic pathway (Figure 6d). The underlying mechanism appears to be the downregulation of ANGPT2, which suppressed the TIE2/PI3K/AKT signaling pathway. This inhibitory effect was further amplified following HMME-PDT treatment, ultimately enhancing HMME-PDT sensitivity and suppressing pathological angiogenesis in R183Q cells (Figure 6e).

## 4 Discussion

**FIGURE 7:**
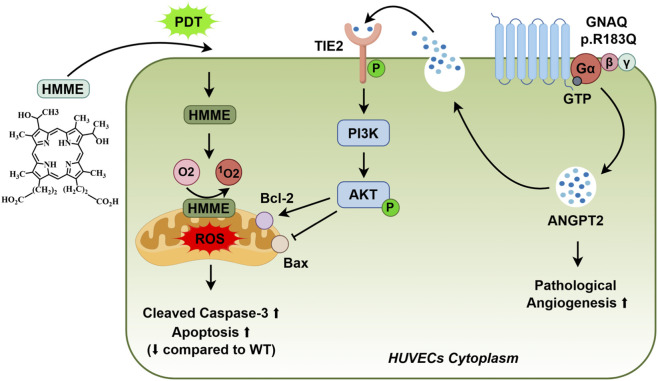
Endothelial *GNAQ* p. R183Q Mutation Confers HMME-PDT Resistance and Drives Pathological Angiogenesis via the ANGPT2/TIE2/PI3K/AKT P.athway.

In this study, we successfully established *GNAQ* p. R183Q mutant ECs and, for the first time, employed them to investigate the responsiveness of HMME-PDT treatment. The p. R183Q mutation promoted pathological angiogenesis and conferred resistance to HMME-PDT-induced mitochondrial apoptosis via activation of the ANGPT2/TIE2/PI3K/AKT signaling pathway (Figure 7). This dual effect facilitated the persistence of abnormal vascular networks, thereby limiting the therapeutic efficacy of HMME-PDT in PWS treatment. Notably, the combination of si-ANGPT2 effectively counteracted these effects by inhibiting pathological angiogenesis and overcoming HMME-PDT resistance. This strategy provides a potential foundation for future therapeutic development, pending further *in vivo* validation.

To model the pathological state of PWS, we employed lentiviral-mediated gene modification to alter the gene expression profile of ECs, thereby amplifying the phenotypic changes driven by the *GNAQ* p. R183Q mutation. This approach has been widely utilized in vascular anomaly research. For instance, overexpression of *GNAQ* p. R183Q in ECs followed by transplantation into nude mice has been shown to successfully recapitulate the characteristics of CMs, including vascular dilation, and is commonly used in mechanistic studies of PWSs and SWS(Nasim et al., 2024; Huang et al., 2023; 2022). Similarly, lentiviral-mediated overexpression of hotspot mutations such as *TIE2* p. L914F or *PIK3CA* p. H1047R in ECs has been demonstrated to reproduce the pathological features of venous malformations (VM) in mouse models (Jauhiainen et al., 2023; Li et al., 2019; Boscolo et al., 2015). However, this approach has clear limitations. In PWS, the *GNAQ* mutation exists as a somatic mosaic variant, and the mutation frequency varies widely, ranging from 1% to nearly 20% in affected tissues (Nguyen et al., 2019). Moreover, previous studies have demonstrated a correlation between mutation frequency and disease severity, suggesting that higher mutation burdens may contribute to more pronounced vascular abnormalities (Wetzel-Strong et al., 2023). Therefore, while lentiviral-mediated overexpression of the mutant gene alone does not fully recapitulate the genetic background of the disease, it remains a valuable tool for investigating its pathogenic mechanisms.

Our findings confirm that the *GNAQ* p. R183Q mutation induces ANGPT2 overexpression, which is consistent with numerous previous studies (Wetzel-Strong et al., 2023; Huang et al., 2022). The R183 residue, located within the GTP-binding pocket, plays a critical role in GTPase activity. The p. R183Q mutation reduces GDP-binding affinity, decreases GTPase activity, and results in sustained activation of the Gαq protein (Martins et al., 2017). This activation triggers the downstream PLCβ signaling pathway, which in turn promotes ANGPT2 expression (Huang et al., 2022). ANGPT2, a pivotal growth factor in the ANG/TIE signaling pathway, is essential for angiogenesis, vascular remodeling, and endothelial homeostasis (Akwii et al., 2019). The regulation of ECs is highly context-dependent. Under physiological conditions, ANGPT2 is stored within Weibel–Palade bodies in ECs. However, under pathological conditions or in response to external stimuli, ANGPT2 is rapidly released via exocytosis and exerts autocrine effects on endothelial function (Chen-Li et al., 2024). Upon secretion, ANGPT2 binds to the TIE2 receptor, functioning as either an agonist or antagonist depending on the microenvironmental context (Saharinen et al., 2017). *In vitro* studies have demonstrated that ANGPT2 overexpression enhances EC migration, promotes angiogenesis, and activates the phosphorylated TIE2 signaling pathway, thereby increasing EC survival (Felcht et al., 2012). Notably, these effects closely mirror the phenotypic characteristics observed in *GNAQ* p. R183Q mutant cells, suggesting that ANGPT2 overexpression may be a key driver of these pathological behaviors.

In this study, we found that elevated ANGPT2 phosphorylates and activates the TIE2 receptor, thereby triggering anti-apoptotic effects via the downstream AKT signaling pathway. Extensive studies have established that AKT suppresses apoptosis through multiple interconnected pathways. First, AKT directly phosphorylates pro-apoptotic proteins, such as Bax at Ser184, inducing conformational changes that inhibit their apoptotic activity (Xu et al., 2023). Additionally, AKT enhances the anti-apoptotic function of Bcl-2 family members, such as Bcl-2 and Bcl-xL, through phosphorylation (Cao et al., 2024). Furthermore, AKT stimulates the mTORC1 pathway, upregulating antioxidant enzymes such as superoxide dismutase (SOD) to mitigate oxidative stress-induced mitochondrial damage (Jaiswal et al., 2022). Beyond apoptosis, autophagy has been implicated in cellular responses to HMME-PDT treatment (Shi et al., 2024). Future studies should investigate whether the *GNAQ* p. R183Q mutation and AKT signaling modulate this process.

PWSs and SWS skin lesions often require multiple rounds of treatment, yet many patients exhibit suboptimal therapeutic responses (Sabeti et al., 2021; Nguyen et al., 2023). To address this challenge, combining HMME-PDT with anti-angiogenic therapy has been proposed as a promising approach, particularly for patients with nodular and hypertrophic lesions. Several research teams have explored the use of topical anti-angiogenic agents in combination with PDL for the treatment of PWSs, including rapamycin (Artzi et al., 2020; Fallahi et al., 2021; Doh et al., 2017; Marqués et al., 2015), timolol (Passeron et al., 2014), axitinib (Gao et al., 2015), imiquimod (Tremaine et al., 2012), and bosentan (Taquin et al., 2016). However, despite these efforts, the therapeutic outcomes have not been as significant as expected (Chou et al., 2024). Future research may need to focus on developing or identifying novel targeted therapies for PWSs. As a key molecule mediating vascular abnormalities in *GNAQ* p. R183Q-mutant ECs, ANGPT2 represents a potential therapeutic target. In our study, siRNA-mediated ANGPT2 inhibition, combined with HMME-PDT, significantly enhanced therapeutic efficacy *in vitro* by promoting EC apoptosis and reducing angiogenesis. This dual effect was achieved via inhibition of the ANGPT2/TIE2/PI3K/AKT pathway, highlighting a potential targeted strategy for PWSs. Anti-angiogenic therapy combined with PDT has been applied in other vascular diseases, such as age-related macular degeneration (AMD) and polypoidal choroidal vasculopathy (PCV). Multiple randomized controlled trials (RCTs) have demonstrated that PDT combined with Vascular Endothelial Growth Factor (VEGF)-targeting agents, such as ranibizumab and aflibercept, significantly reduces pathological vascular formation in the retina and provides substantial benefits for refractory patients (Gao et al., 2018; Cheung et al., 2017). This approach has been widely adopted in clinical practice. Currently, the second-generation anti-angiogenic agent Faricimab, which simultaneously targets VEGF and ANGPT2, has completed phase III clinical trials for AMD, demonstrating significant efficacy (ClinicalTrials.gov: NCT03823287, NCT03823300) (Khanani et al., 2024, p. 2). However, whether its combination with PDT could provide additional therapeutic benefits remains to be further investigated. In conclusion, these findings provide new insights, suggesting that PDT combined with anti-angiogenic therapy, particularly targeting ANGPT2, could be a highly promising treatment strategy for PWSs.

Despite the promising results of this study, several limitations must be considered. The primary limitation is that this study was conducted solely *in vitro*, making it difficult to fully simulate the actual conditions of PWSs patients carrying the *GNAQ* p. R183Q mutation. This is due to the pediatric nature of the study population at our center, which raises ethical concerns, as well as the fact that PWS primarily affects the face and neck, where genetic sampling may impact appearance. Future research should apply these findings *in vivo* or in clinical settings to assess their clinical consistency and feasibility. In particular, the recently developed endothelial-specific *GNAQ* R183Q knock-in mouse model by Smits et al. (Smits et al., 2025), which recapitulates CMs phenotypes in skin and brain vasculature while avoiding embryonic lethality, offers a promising platform for vivo evaluation.

In conclusion, our study demonstrates that the endothelial *GNAQ* p. R183Q mutation leads to increased expression of ANGPT2. Elevated ANGPT2 not only promotes pathological angiogenesis but also mediates endothelial resistance to HMME-PDT through activation of the TIE2/PI3K/AKT pathway. Targeting ANGPT2 in combination with HMME-PDT achieves a dual effect by simultaneously inhibiting angiogenesis and enhancing apoptosis. Currently, no targeted therapies are available for patients with PWSs. Our findings suggest that ANGPT2 could serve as a critical therapeutic target for drug development, potentially offering novel treatment strategies for these patients.

## Data Availability

The original contributions presented in the study are included in the article/Supplementary Material, further inquiries can be directed to the corresponding authors.
