# Supplementary material for: Endothelial GNAQ p.R183Q mutation confers hemoporfin-mediated photodynamic therapy resistance and drives pathological angiogenesis via the angiopoietin-2/TIE2/PI3K/AKT pathway
Source: Front Cell Dev Biol. 2025 Aug 18;13:1622961. doi: 10.3389/fcell.2025.1622961 (PMC12411732; doi:10.3389/fcell.2025.1622961)
Supplement: Supplementary file 1 [file DataSheet1.docx]

Supplementary Material

# *GNAQ* p.R183Q Mutant Sequence

**Reference Sequence**: NM_002072 (R183Q)

**Mutation**: *GNAQ* p.R183Q c.548G>A

**RNA Sequence:**

AtgactctggagtccatcatggcgtgctgcctgagcgaggaggccaaggaagcccggcggatcaacgacgagatcgagcggcagctccgcagggacaagcgggacgcccgccgggagctcaagctgctgctgctcgggacaggagagagtggcaagagtacgtttatcaagcagatgagaatcatccatgggtcaggatactctgatgaagataaaaggggcttcaccaagctggtgtatcagaacatcttcacggccatgcaggccatgatcagagccatggacacactcaagatcccatacaagtatgagcacaataaggctcatgcacaattagttcgagaagttgatgtggagaaggtgtctgcttttgagaatccatatgtagatgcaataaagagtttatggaatgatcctggaatccaggaatgctatgatagacgacgagaatatcaattatctgactctaccaaatactatcttaatgacttggaccgcgtagctgaccctgcctacctgcctacgcaacaagatgtgcttagagttcAagtccccaccacagggatcatcgaatacccctttgacttacaaagtgtcattttcagaatggtcgatgtagggggccaaaggtcagagagaagaaaatggatacactgctttgaaaatgtcacctctatcatgtttctagtagcgcttagtgaatatgatcaagttctcgtggagtcagacaatgagaaccgaatggaggaaagcaaggctctctttagaacaattatcacatacccctggttccagaactcctcggttattctgttcttaaacaagaaagatcttctagaggagaaaatcatgtattcccatctagtcgactacttcccagaatatgatggaccccagagagatgcccaggcagcccgagaattcattctgaagatgttcgtggacctgaacccagacagtgacaaaattatctactcccacttcacgtgcgccacagacaccgagaatatccgctttgtctttgctgccgtcaaggacaccatcctccagttgaacctgaaggagtacaatctggtctaa

**Protein Sequence:** MTLESIMACCLSEEAKEARRINDEIERQLRRDKRDARRELKLLLLGTGESGKSTFIKQMRIIHGSGYSDEDKRGFTKLVYQNIFTAMQAMIRAMDTLKIPYKYEHNKAHAQLVREVDVEKVSAFENPYVDAIKSLWNDPGIQECYDRRREYQLSDSTKYYLNDLDRVADPAYLPTQQDVLRVQVPTTGIIEYPFDLQSVIFRMVDVGGQRSERRKWIHCFENVTSIMFLVALSEYDQVLVESDNENRMEESKALFRTIITYPWFQNSSVILFLNKKDLLEEKIMYSHLVDYFPEYDGPQRDAQAAREFILKMFVDLNPDSDKIIYSHFTCATDTENIRFVFAAVKDTILQLNLKEYNLV

In the sequence shown, the *GNAQ* p.R183Q mutation is indicated by the red-highlighted region. This mutation involves a single nucleotide substitution, where guanine (G) is replaced by adenine (A), resulting in the amino acid change from arginine (R) to glutamine (Q) at position 183 of the Gαq protein.

# Supplementary Figures and Tables


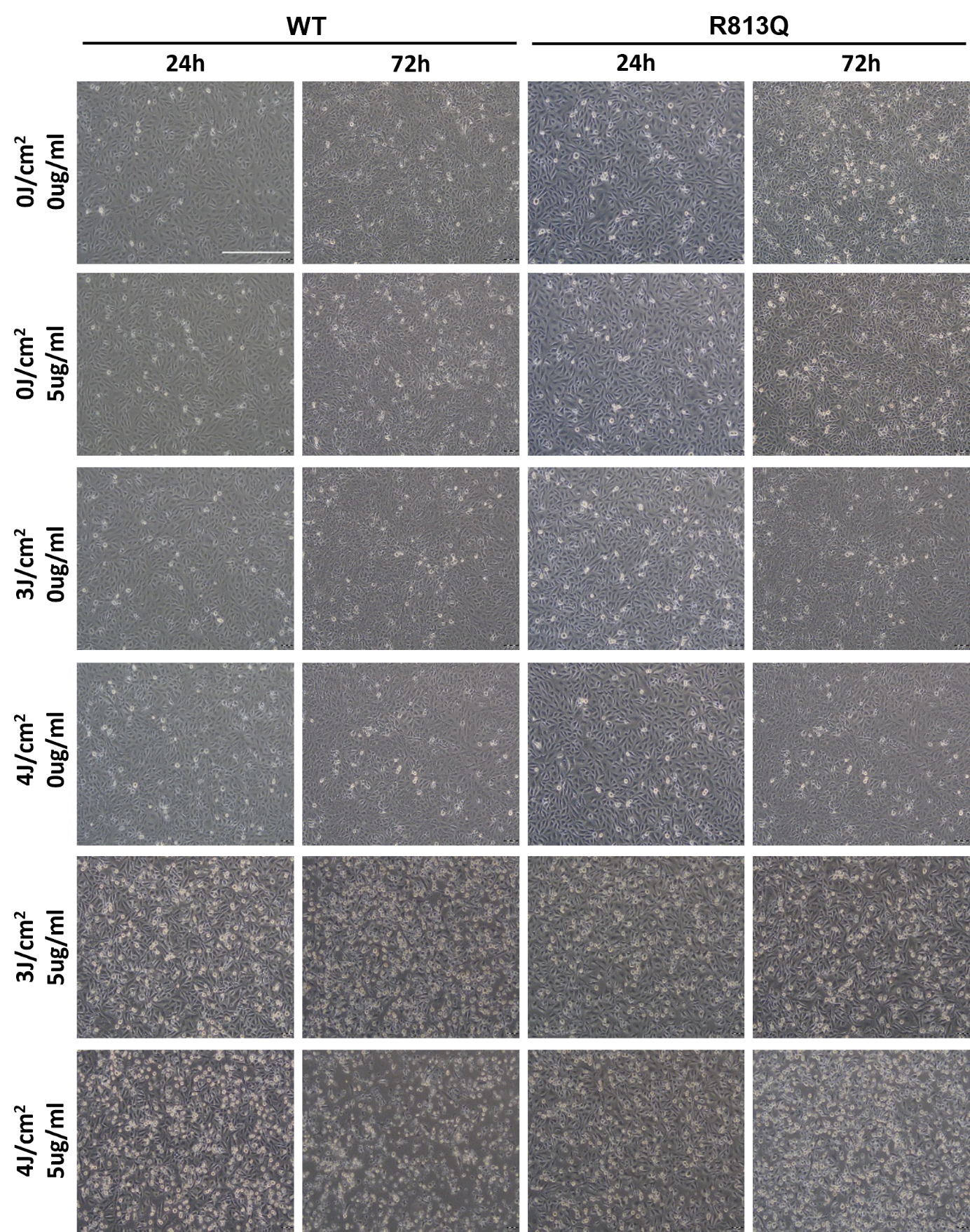


**Fig. S1** Representative Bright-Field Images of WT and R183Q Cells Under Different HMME-PDT Treatment Conditions. Representative bright-field images of WT and R183Q cells subjected to HMME-PDT under different treatment conditions, including HMME concentrations of 0 μg/ml or 5 μg/ml, observed at 24 or 72 hours post-PDT, and exposed to energy densities of 0 J/cm², 3 J/cm², or 4 J/cm². These images illustrate cellular responses across varying photosensitizer concentrations, time points, and irradiation intensities. (n = 3, Scale bars: 500 μm).


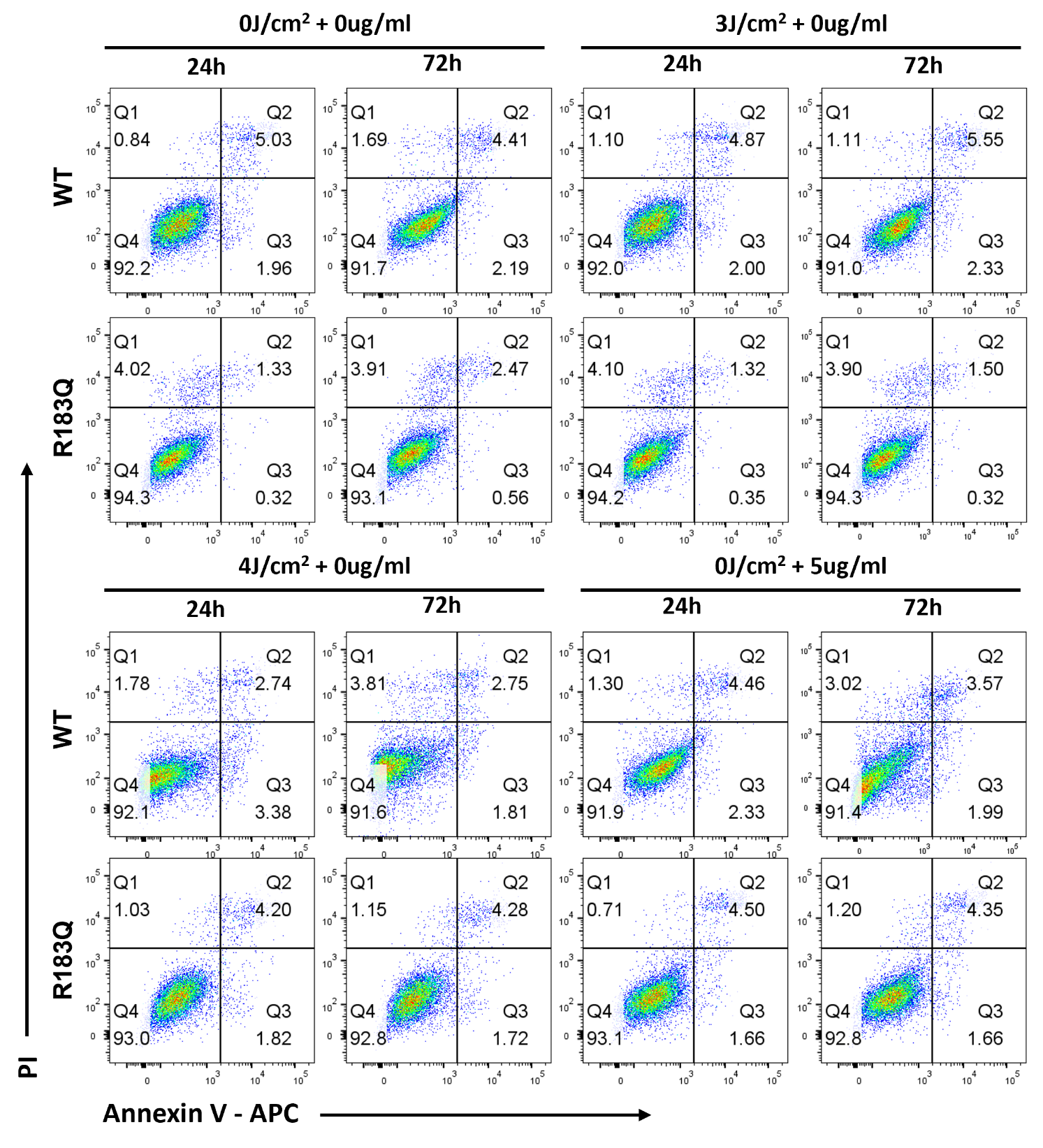


**Fig. S2** Flow Cytometric Analysis of Apoptotic Responses Under Control Conditions. Annexin V/PI flow cytometric comparison of apoptotic responses in WT and R183Q cells following HMME-PDT at the control conditions, evaluated at 24 and 72 hours post-PDT. Control conditions included untreated cells, cells treated with HMME alone (5ug/ml), and cells exposed to light alone (3J/cm^2^ or 4J/cm^2^). (n = 3).


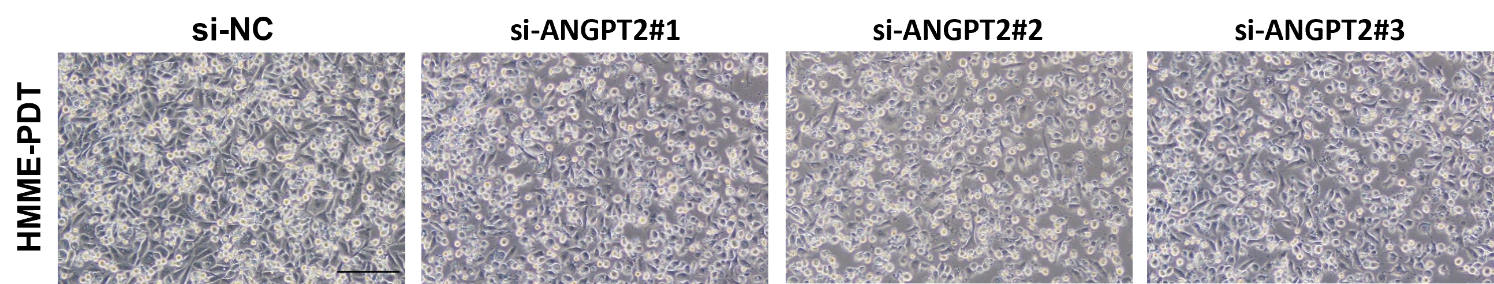


**Fig. S3** Representative bright-field images of R183Q cells post-PDT following siRNA-mediated ANGPT2 knockdown. (scale bars: 200 μm).

**Table S1. Primers used for RT-qPCR**

| Gene name | Sequence (5'→3') | |
| --- | --- | --- |
| *GAPDH* | Forward | GACTCATGACCACAGTCCATGC |
|  | Reverse | AGAGGCAGGGATGATGTTCTG |
| *GNAQ* | Forward | TCAACGACGAGATCGAGCG |
|  | Reverse | ACTCTCTCCTGTCCCGAGC |
| *CASP3* | Forward | CATGGAAGCGAATCAATGGACT |
|  | Reverse | CTGTACCAGACCGAGATGTCA |
| *BCL-2* | Forward | GGTGGGGTCATGTGTGTGG |
|  | Reverse | CGGTTCAGGTACTCAGTCATCC |
| *BAX* | Forward | CCCGAGAGGTCTTTTTCCGAG |
|  | Reverse | CCAGCCCATGATGGTTCTGAT |
| *ANGPT2* | Forward | ACTCAGCTAAGGACCCCACTGTTG |
|  | Reverse | TGTCCACCCGCCTCCTCCAG |
| *TIE2* | Forward | GGGACTTTGCAGGAGAACTGG |
|  | Reverse | AAATGCTGGGTCCGTCTCCA |

**Table S2. siRNA sequences**

| Target Gene | siRNA ID | Sequences (5'→3') |
| --- | --- | --- |
| *ANGPT2* | si-ANGPT2#1 | CAAGUGAAGAACUCAAUUA |
|  |  | UAAUUGAGUUCUUCACUUG |
|  | si-ANGPT2#2 | GAUCUCAUGGAGACAGUUA |
|  |  | UAACUGUCUCCAUGAGAUC |
|  | si-ANGPT2#3 | GGCAUCUACACGUUAACAU |
|  |  | AUGUUAACGUGUAGAUGCC |
| Control siRNA | si-NC | UUCUCCGAACGUGUCACGU |
|  |  | ACGUGACACGUUCGGAGAA |
